# Nutritional supplements for migraine prevention: evaluating evidence sufficiency and effect modifiers through network meta-analysis, trial sequential analysis, and meta-regression

**DOI:** 10.3389/fnut.2026.1911863

**Published:** 2026-08-24

**Authors:** Jingjing Peng, Ling Zhou, Yuxian Chen, Jian Li, Rui Zhang

**Affiliations:** Department of Pharmacy, West China Hospital, Sichuan University, Chengdu, Sichuan Province, China

**Keywords:** meta-regression, migraine prevention, network meta-analysis, nutritional supplements, treatment ranking, trial sequential analysis

## Abstract

**Background:**

This network meta-analysis (NMA) integrated trial sequential analysis (TSA) limited to eligible direct pairwise comparisons and network meta-regression to compare efficacy and safety of nutritional supplements for migraine prevention, assess evidence maturity and identify potential effect modifiers.

**Methods:**

We retrieved randomized controlled trials (RCTs) of nutritional supplements for adult migraineurs from four databases up to January 2025. Primary outcomes were migraine frequency, duration and severity; adverse events were the secondary outcome. Frequentist random-effects NMA calculated mean differences (MD) for continuous indicators and odds ratios (OR) for adverse events with 95% confidence intervals (CIs). Treatment rankings were assessed using rank probabilities and the surface under the cumulative ranking curve but were interpreted as exploratory. Inconsistency was tested where network structure allowed valid evaluation. Network meta-regression detected between-study heterogeneity, TSA was restricted to eligible direct pairwise comparisons and the CINeMA framework evaluated overall evidence certainty.

**Results:**

Fourteen RCTs (791 participants) formed a six-node network: vitamins, magnesium, coenzyme Q10, probiotics, vitamin-probiotic combinations and placebo. For migraine frequency, probiotics produced the largest frequency reduction vs. placebo (MD = −2.60 attacks/month, 95% CI − 3.96 to −1.20; 72% top-rank probability), followed by vitamin-probiotics (MD = −1.92, 95% CI: −3.12 to −0.73), coenzyme Q10 (MD = −1.91, 95% CI: −2.51 to −1.00) and vitamins (MD = −1.85, 95% CI: −2.65 to −1.05). Magnesium showed non-significant benefits (MD = −0.82, 95% CI − 2.03 to 0.13), and no active supplements differed statistically from each other. No intervention significantly shortened migraine duration; vitamin-probiotics ranked first (MD = −4.68 h/attack, 95% CI − 9.76 to 0.15). Only magnesium significantly lowered severity (MD = −3.56 points on the 0–10 VAS, 95% CI − 6.50 to −0.52; 85% top-rank probability). Treatment duration correlated with frequency improvements (coefficient = −0.09, 95% CI − 0.17 to −0.02, *p* = 0.01). Only one probiotic-placebo trial precluded definitive TSA evidence conclusions for probiotics, and CINeMA graded most comparisons as moderate-certainty evidence.

**Conclusion:**

Probiotics best reduced migraine frequency yet lacked statistical superiority over other supplements via indirect placebo comparisons. Only magnesium alleviated migraine severity, while no supplement significantly shortened attack duration. Treatment rankings remain exploratory. Large standardized head-to-head RCTs are required for validation.

**Systematic review registration:**

https://www.crd.york.ac.uk/PROSPERO/view/CRD420251182820, CRD420251182820.

## 1 Introduction

Migraine is a prevalent and debilitating neurological disorder affecting approximately 12% of the global population, with a disproportionate impact on women during their reproductive years (1). The condition is characterized by recurrent episodes of moderate to severe headache, often accompanied by nausea, vomiting, photophobia, and phonophobia, resulting in substantial functional disability and reduced quality of life (2). The economic burden of migraine is considerable, with annual direct and indirect costs estimated at billions of dollars worldwide due to healthcare utilization, reduced productivity, and absenteeism (3).

Current pharmacological approaches for migraine prevention include beta-blockers, anticonvulsants, calcium channel blockers, and antidepressants, which demonstrate varying degrees of efficacy but are often associated with significant adverse effects and contraindications (4). These limitations have prompted increasing interest in complementary and alternative therapeutic strategies, particularly nutritional supplements, which may offer comparable efficacy with improved tolerability profiles (5).

Several biological mechanisms support the potential role of nutritional supplements in migraine pathophysiology. Magnesium deficiency has been implicated in cortical spreading depression, the proposed neurobiological substrate of migraine aura, and magnesium supplementation may stabilize neuronal membranes and modulate neurotransmitter release (6). Coenzyme Q10, a mitochondrial cofactor essential for cellular energy production, addresses the mitochondrial dysfunction hypothesis of migraine, where impaired energy metabolism may contribute to neuronal hyperexcitability (7). B-vitamins, particularly riboflavin, folate, and cobalamin, participate in homocysteine metabolism and may influence vascular function and inflammation, both relevant to migraine pathogenesis (8).

Emerging evidence suggests that the gut-brain axis may play a crucial role in migraine development, with alterations in intestinal microbiota composition potentially contributing to neuroinflammation and neuropeptide dysregulation (9). This has led to investigations into probiotic supplementation as a novel therapeutic approach, with preliminary studies demonstrating promising results in reducing migraine frequency and severity (10).

Despite the growing body of evidence supporting various nutritional interventions, direct head-to-head comparisons among different supplements are scarce, making it challenging for clinicians to make evidence-based treatment decisions. Traditional pairwise meta-analyses have examined individual supplements in isolation but cannot provide comparative effectiveness rankings across multiple interventions (11). Network meta-analysis enables simultaneous comparison of multiple treatments by synthesizing direct and indirect evidence, although the reliability of such comparisons depends on transitivity, network connectivity, and the availability of direct evidence (12).

Previous systematic reviews have examined specific nutritional supplements individually, with magnesium supplementation showing modest benefits in migraine prevention (13), riboflavin demonstrating significant reductions in migraine frequency (14), and coenzyme Q10 exhibiting promise in pediatric populations (15). However, the relative effectiveness of these interventions remains unclear, and combined approaches using multiple supplements have received limited investigation.

More recently, several meta-analytic efforts have advanced our understanding of nutritional supplements in migraine prevention. Talandashti et al. conducted a systematic review and dose–response meta-analysis of 22 randomized controlled trials, demonstrating that magnesium, coenzyme Q10, and riboflavin each significantly reduced migraine frequency and severity, and further explored dose-dependent relationships using GRADE-assessed evidence (16). Ketata and Ellouz performed a network meta-analysis specifically comparing the efficacy of various nutraceuticals on migraine symptom relief, reporting that coenzyme Q10, vitamin D3, curcumin, and cinnamon ranked highest for reducing different migraine characteristics (17).

Additionally, Tseng et al. conducted a large-scale network meta-analysis of 40 randomized controlled trials encompassing 6,616 participants, comparing various dosages of omega-3 fatty acids with FDA-approved pharmacological agents, and found that high-dose EPA/DHA supplementation demonstrated superior efficacy for migraine prophylaxis (18).

While these studies have made important contributions, several gaps remain. In contrast to pairwise meta-analyses examining individual supplements separately, the present study uses network meta-analysis to compare multiple supplement categories within a single analytical framework. TSA was restricted to eligible direct pairwise comparisons to assess the maturity of accumulated direct evidence under prespecified assumptions rather than to validate indirect network estimates. Network meta-regression was used to explore whether intervention duration, baseline migraine frequency, and demographic characteristics were associated with between-study heterogeneity. Our network also included probiotics and vitamin-probiotic combination interventions as separate categories. These methodological features provide an integrated evidence framework combining comparative estimates, exploratory treatment rankings, direct-evidence maturity, and potential study-level effect modifiers.

The heterogeneity in study populations, intervention protocols, outcome measures, and follow-up durations across existing trials further complicates the interpretation of available evidence. Standardized approaches to assess migraine-related outcomes, including the Headache Impact Test (HIT-6), Migraine Disability Assessment (MIDAS), and visual analog scales for pain intensity, have been variably implemented across studies (19).

Furthermore, safety considerations are paramount in migraine prevention, particularly given the chronic nature of the condition requiring long-term treatment. While nutritional supplements are generally perceived as having favorable safety profiles compared to conventional pharmacological agents, systematic evaluation of adverse events across different interventions is essential for informed clinical decision-making.

We therefore conducted a network meta-analysis to evaluate the comparative effectiveness of nutritional supplements for migraine prevention, apply TSA exclusively to eligible direct pairwise evidence, explore potential study-level effect modifiers using network meta-regression, assess exploratory treatment rankings, and evaluate the safety profiles of the included interventions.

## 2 Methods

### 2.1 Protocol registration and reporting standards

This network meta-analysis was conducted according to the Preferred Reporting Items for Systematic Reviews and Meta-Analyses extension for Network Meta-Analyses (PRISMA-NMA) guidelines (Supplementary file 1). The study protocol was prospectively registered in the International Prospective Register of Systematic Reviews (PROSPERO: CRD420251182820).

### 2.2 Eligibility criteria

#### 2.2.1 Study design

We included randomized controlled trials comparing an orally administered nutritional supplement or nutritional-supplement combination with placebo, usual care, no treatment, or another eligible nutritional intervention. Cross-over trials were eligible if adequate washout periods were implemented. Quasi-randomized studies, observational studies, and single-arm trials were excluded. Open-label add-on studies in which the effect of the nutritional supplement could not be separated from an active pharmacological co-intervention were excluded from the primary network.

#### 2.2.2 Participants

Eligible studies enrolled adult participants (≥18 years) with a clinical diagnosis of episodic migraine according to International Classification of Headache Disorders (ICHD) criteria or physician diagnosis based on characteristic clinical features. Studies including participants with chronic migraine (≥15 headache days per month), medication overuse headache, or other primary headache disorders were excluded unless migraine-specific data could be extracted separately.

#### 2.2.3 Interventions

We included studies investigating any nutritional supplement administered orally for migraine prevention, including vitamins (B-complex, riboflavin, folate, vitamin D), minerals (magnesium, calcium), coenzymes (coenzyme Q10), probiotics, omega-3 fatty acids, herbal supplements, and combination formulations. Minimum treatment duration of 4 weeks was required. Interventions administered via non-oral routes or as part of comprehensive lifestyle programs were excluded.

##### 2.2.3.1 Intervention node classification

Because only a limited number of trials evaluated each individual supplement, interventions were grouped into clinically related categories to maintain network connectivity. The “vitamins” node encompassed riboflavin, vitamin D3, and alpha-lipoic acid, which were grouped as micronutrient or vitamin-like cofactors involved in cellular metabolism and oxidative-stress pathways. The “probiotics” node included multispecies probiotic formulations based on their shared mechanism of gut-microbiota modulation. The “coenzyme Q10” node included studies using coenzyme Q10 as a single-agent intervention. The “magnesium” node included studies using different oral magnesium formulations. The “vitamin plus probiotics” node included studies combining vitamin supplementation with probiotic supplementation.

This grouping strategy was pragmatic and does not imply that interventions within the same node are pharmacologically interchangeable. Individual supplements and formulations may differ in biological mechanisms, bioavailability, optimal dosage, and clinical effects. Results should therefore be interpreted at the broad supplement-category level rather than extrapolated to a particular formulation or dose.

#### 2.2.4 Comparators

Eligible comparators included placebo (inert substance matching the appearance of active treatment), usual care (continuation of participants’ existing migraine management without prophylactic supplements, which could include acute medications and lifestyle advice), no treatment, or other active nutritional supplements. Studies evaluating a supplement solely as an open-label add-on to an active pharmacological treatment were excluded when the supplement effect could not be appropriately mapped to the intervention network.

#### 2.2.5 Outcomes

Primary outcomes comprised migraine frequency (attacks per month), migraine duration (hours per attack), and migraine severity assessed using validated scales (visual analog scale, numerical rating scale, or categorical measures). Secondary outcomes included adverse events (any reported adverse effects), quality of life measures, and functional disability scores. For studies reporting outcomes at multiple time points, we prioritized data from 12 weeks of treatment, or the closest available time point between 8 and 24 weeks. When studies reported multiple measures of the same outcome, we selected the most commonly reported measure across studies to maintain network connectivity.

### 2.3 Information sources and search strategy

Comprehensive literature searches were conducted in MEDLINE (via PubMed), Embase, Cochrane Central Register of Controlled Trials (CENTRAL), and Web of Science from database inception to January 31, 2025. The complete search strategy for PubMed/MEDLINE is provided in Supplementary file 2. Search strategies combined controlled vocabulary terms and free-text keywords related to migraine, nutritional supplements, and randomized controlled trials, without language restrictions.

Additional sources were systematically searched to reduce the potential for publication bias. Reference lists of all included studies, relevant systematic reviews, and clinical practice guidelines were hand-searched for additional eligible trials. Trial registries, including ClinicalTrials.gov and the WHO International Clinical Trials Registry Platform (ICTRP), were searched for completed but unpublished studies, and study authors were contacted when registry entries indicated completed trials without corresponding publications. Conference abstracts from major headache society meetings (International Headache Congress, American Headache Society Annual Scientific Meeting, European Headache Federation Congress) and nutrition society meetings (American Society for Nutrition, European Society for Clinical Nutrition and Metabolism) from 2015 to 2025 were reviewed for relevant presentations. Dissertations and theses were searched through ProQuest Dissertations & Theses Global. No eligible unpublished trials or gray literature sources meeting inclusion criteria were identified through these supplementary searches.

### 2.4 Study selection and data extraction

Two reviewers (PJ and ZL) independently screened titles and abstracts using predefined eligibility criteria, followed by full-text review of potentially eligible studies. Disagreements were resolved through discussion or consultation with a third reviewer (ZR). Inter-rater agreement was calculated using Cohen’s kappa statistic.

Data extraction was performed independently by two reviewers (PJ and ZL) using standardized, piloted forms capturing study characteristics (design, setting, duration), participant demographics (age, sex, migraine characteristics), intervention details (type, dose, frequency, duration), outcome measures and time points, and methodological features relevant to risk of bias assessment. Discrepancies were resolved through discussion with reference to original publications. When data were unclear or missing, we contacted study authors via email. For studies reporting only median and interquartile range, we estimated mean and standard deviation using established methods. All assumptions were documented and explored in sensitivity analyses.

### 2.5 Risk of bias assessment

Risk of bias was assessed independently by two reviewers (PJ and ZL) using the Cochrane Risk of Bias 2 (RoB 2) tool at the outcome level for each primary outcome and for adverse events, evaluating randomization process, deviations from intended interventions, missing outcome data, measurement of outcomes, and selective reporting. Each domain was rated as low risk, some concerns, or high risk, with overall study risk determined by the highest individual domain rating. Disagreements were resolved through discussion or third-party adjudication (ZR).

### 2.6 Data synthesis and statistical analysis

#### 2.6.1 Network geometry

Network plots were constructed to visualize treatment comparisons and assess network connectivity. Node size represented the number of participants receiving each intervention, while edge thickness reflected the number of direct comparisons.

#### 2.6.2 Network meta-analysis

We performed frequentist network meta-analysis using a random-effects model within a multivariate framework. For continuous outcomes, including migraine frequency, duration, and severity, we calculated mean differences with 95% confidence intervals using the original measurement scales. Migraine frequency was expressed as attacks per month, migraine duration as hours per attack, and migraine severity as points on the visual analog scale. Mean differences were selected because the studies contributing to each outcome used sufficiently comparable measurement scales, allowing direct clinical interpretation. For adverse events, odds ratios with 95% confidence intervals were calculated.

##### 2.6.2.1 Assessment of transitivity

The transitivity assumption was assessed before conducting the network meta-analysis by examining the distributions of potential clinical and methodological effect modifiers across direct comparisons. These factors included mean age, proportion of female participants, baseline migraine frequency, intervention duration, and study quality. Mean age ranged from 33.2 to 41.8 years, the proportion of female participants ranged from 72 to 89%, baseline migraine frequency ranged from 3.2 to 7.8 attacks per month, and intervention duration ranged from 8 to 24 weeks. No statistically significant differences were detected across comparison groups. These findings supported the clinical plausibility of transitivity, although the small number of studies and the possibility of unmeasured effect modifiers limited the strength of this assessment.

#### 2.6.3 Heterogeneity and inconsistency assessment

Statistical heterogeneity was assessed using the I^2^ statistic and tau-squared values. Network inconsistency was evaluated using the design-by-treatment interaction model for global inconsistency and node-splitting analysis only where closed loops provided both direct and indirect evidence.

#### 2.6.4 Treatment rankings

Treatment rankings were assessed using rank probabilities and surface under the cumulative ranking curve values. The probability of ranking first and the SUCRA value were treated as distinct measures and were not used interchangeably. Rankings were interpreted together with effect estimates, confidence intervals, direct-evidence availability, and certainty assessments. A favorable ranking was not interpreted as statistically robust superiority when the corresponding confidence interval was wide or crossed the null value.

#### 2.6.5 Subgroup and sensitivity analyses

Predefined subgroup analyses examined intervention duration, baseline migraine frequency, and study quality. Sensitivity analyses assessed the influence of risk of bias, blinding, alternative effect measures, publication period, and individual studies.

#### 2.6.6 Network meta-regression

Network meta-regression was conducted to explore potential study-level effect modifiers. Prespecified covariates included mean age, proportion of female participants, baseline migraine frequency, intervention duration, publication year, and sample size.

#### 2.6.7 Trial sequential analysis

Trial sequential analysis was restricted to direct pairwise comparisons between a specific nutritional supplement and placebo or control. Indirect and mixed estimates generated by the network meta-analysis were not entered into TSA because TSA methodology has not been validated for network-derived indirect comparisons. Comparisons represented by only one direct trial were not used to draw TSA-based evidence-sufficiency conclusions because TSA is intended to evaluate cumulative evidence across trials.

For migraine frequency, the prespecified clinically relevant difference was set at 1.0 attack per month. For severity, the prespecified difference was 1.5 points on the 10-point visual analog scale, and for duration it was 3.0 h per attack. These thresholds were described as prespecified clinically relevant differences rather than established migraine-specific MCIDs when direct anchor-based validation was unavailable (20).

TSA used a two-sided alpha of 0.05, statistical power of 80%, and diversity-adjusted required information sizes. Cumulative Z-curves were compared with conventional significance boundaries and O’Brien–Fleming-type trial sequential monitoring boundaries. TSA findings were interpreted cautiously because they were sensitive to the assumed intervention effect, heterogeneity, type I and type II error rates, and the number of contributing trials. TSA findings apply only to the corresponding direct comparison and cannot be extrapolated to indirect network estimates or treatment rankings.

#### 2.6.8 Assessment of evidence certainty

Evidence certainty was evaluated using the Confidence in Network Meta-Analysis (CINeMA) framework, assessing within-study bias, reporting bias, indirectness, imprecision, heterogeneity, and incoherence for each pairwise comparison and outcome. Evidence was classified as high, moderate, low, or very low certainty.

#### 2.6.9 Assessment of small-study effects and publication bias

Potential small-study effects and publication bias were explored using comparison-adjusted funnel plots, Egger’s regression test, and Begg’s rank-correlation test. Because these formal tests have limited statistical power when the number of contributing studies is small, their results were interpreted cautiously. Nonsignificant results were not considered evidence that publication bias was absent.

#### 2.6.10 Software and statistical packages

All analyses were conducted using R version 4.3.0 with the netmeta, meta, metafor, and ggplot2 packages. TSA was conducted using TSA software version 0.9.5.10 Beta.

## 3 Results

### 3.1 Characteristics of included studies

**Figure 1:**
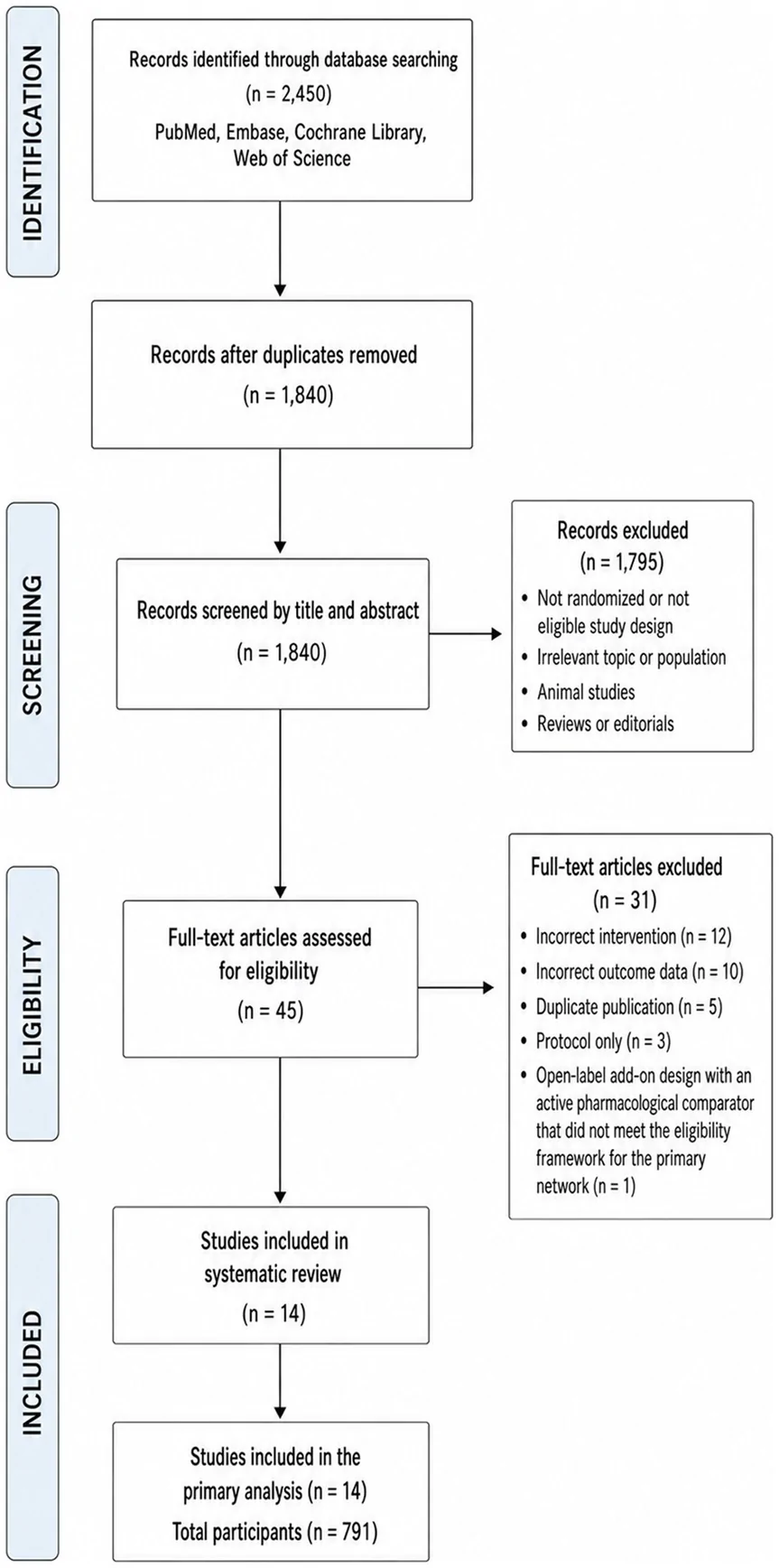
PRISMA 2020 flow diagram. Final network composition: Total participants: 791; Interventions: Magnesium, coenzyme Q10, vitamins, probiotics, vitamin+probiotics combinations; Primary outcomes: Migraine frequency, duration, severity; Study duration range: 8–24 weeks.

The primary analysis included 14 randomized controlled trials involving 791 participants (7, 21–33). Figure 1 summarized the flow chart. Shoeibi et al. 2017 (34) was excluded because its open-label add-on design comparing coenzyme Q10 plus nortriptyline with nortriptyline alone did not meet the eligibility framework applied to the primary network. This study was not included in any quantitative analysis. Ten studies contributed to the migraine-duration analysis, 13 studies involving 693 participants contributed to the migraine-frequency analysis, and 13 studies contributed to the migraine-severity analysis. Table 1 summarizes the characteristics of all included studies.

**Table 1:** The characteristics of the included trials.

Author	Year	Country	Study type	Nutritional Supplements	Control	Sample size		Age (mean years)		Female (%)		Baseline attack frequency/month		Baseline attack duration/h		Patient status	Duration time	Main Endpoint
						IG	CG	IG	CG	IG	CG	IG	CG	IG	CG			
Peikert	1996	Germany	RCT	Magnesium	placebo	43	38	43.8 ± 10.7	47.6 ± 10.0	86	86.8	3.63 ± 1.76	3.66 ± 1.71	NA	NA	Migraine that fulfills the IHS diagnostic criteria (mean attack frequency 3.6 per month)	12 weeks	frequency of attacks/4 weeks duration of attacks(days) severity of attacks (VAS)
Pfaffenrath	1996	Germany	RCT	Magnesium	placebo	35	34	41 ± 12	40 ± 13	97	88	NA	NA	NA	NA	at least 2 years of migraine without aura by IHS criteria; 2 to 6 migraine attacks per month	16 weeks	migraine duration in hours intensity of migraine
Schoenen	1998	Belgium	RCT	riboflavin	placebo	28	26	36.9	35.2	75	80	3.83	3.71	35.42	32.35	met the IHS diagnostic criteria for migraine with or without aura and had a history of migraine of at least 1 year (2 to 8 attacks per month)	16 weeks	attack frequency duration severity
Sándor	2005	Switzerland	RCT	CoQ10	placebo	21	21	37.7 ± 12	39.6 ± 16	85.7	76.2	4.4 ± 1.9	4.4 ± 1.6	7.6 ± 4.8	6.0 ± 3.4	met the IHS diagnostic criteria for migraine with or without aura and had a history of migraine of at least 1 year (2 to 8 attacks per month)	16 weeks	attack frequency mean attack duration Mean attack severity
Magis	2006	Belgium	RCT	Thioctic Acid	placebo	26	18	37.46 ± 13.43	38.94 ± 8.05	84.6	88.9	3.46 ± 1.94	3.06 ± 1.55	7.70 ± 6.58	9.69 ± 5.47	migraineurs with or without aura, fulfilling the diagnostic criteria of the International Classification of Headache Disorders 2nd edition, 2 to 6 attacks per month	16 weeks	attack frequency mean duration of attacks mean severity
Köseoglu	2008	Turkey	RCT	magnesium	placebo	30	10	36.6 ± 9.3	43.5	86.7	90	3.5 ± 8.2	3.5	NA	NA	at least 2 years of migraine without aura according to the criteria of IHS	12 weeks	attack frequency mean severity
Mottaghi	2015	Iran	RCT	Vitamin D	placebo	33	32	32.7 ± 10.6	33.9 ± 11.6	75.8	68.8	8.4 ± 7.6	8.0 ± 6.4	22.9 ± 17.7	26.5 ± 21.7	Confirmation of migraine disease was done by a neurologist.	10 weeks	frequency of attacks/month severity mean attack duration
De Roos	2017	Netherlands	RCT	multispecies probiotic	placebo	31	29	42	38	90.3	96.6	NA	NA	NA	NA	Subjects fulfilling the IHC(ICHD-II) criteria, and who had at least four migraine attacks per month	12 weeks	MIDAS HDI
Dahri	2018	Iran	RCT	CoQ10	placebo	23	22	32.35 ± 6.60	32.32 ± 7.52	100	100	8.47	6.36	11.33	13.02	episodic migraine according to the IHS, and more than 1 year with at least two attacks per month	16 weeks	attacks (in hours) migraine attack frequency attack severity
Gazerani	2019	Denmark	RCT	vitamin D3	placebo	24	24	45.5 ± 12.1	43.7 ± 10.2	75	75	3.00 ± 1.07	3.75 ± 1.98	NA	NA	Migraine diagnosis was based on criteria set by the International Headache Society for headache classification (ICHD-II)	24 weeks	attack frequency per month Attack severity
Martami	2018	Iran	RCT	multispecies probiotic	placebo	22	18	36.27 ± 6.99	39.82 ± 8.11	68.2	72.2	6.59 ± 2.81	6.94 ± 3.30	7.25 ± 3.66	11.39 ± 4.99	based on an expert neurolo- gist headache specialist’s diagnosis, according to the ICHD III criteria (beta version).	10 weeks	Attack severity attack frequency per month Attack duration (hour/attack)
Ghorbani	2019	Iran	RCT	Vitamin D3	placebo	38	36	37 ± 8	38 ± 12	84.2	88.9	6.33 ± 3.73	6.3 ± 3.02	23.12 ± 16.19	21.42 ± 8.43	diagnosis of episodic migraine (EM) defined as having ≤ 15 headache days in a month in addition to having migraine characteristics according to the ICHDIII criteria	12 weeks	attack severity attack frequency per month Attack duration (hours per attack)
Kelishadi	2022	Iran	RCT	alpha-lipoic acid	placebo	42	37	40.28	43.31	100	100	5.723	5.733	36.957	42.872	met the criteria of the International Headache Society (IHS)	12 weeks	headache duration per attack headache severity headache frequency per month
Tirani	2024	Iran	triple-blinded RCT	Probiotic and vitamin D	placebo	36	36	37.44 ± 8.84	37.47 ± 7.90	91.7	88.9	7.76	5.97	23.26	23.1	a history of migraine with or without aura based on the ICHD-3, and experienced more than 2 migraine attacks per month during the 3 preceding months	12 weeks	attack frequency/month, attack duration (hours/month), attack severity

IHS, International Headache Society; IG, Intervention Group; CG, Control Group; VAS, Visual analog scale; HDI, Headache Disability Inventory; MIDAS, Migraine Disability Assessment Scale.

### 3.2 Methodological quality assessment of trials included

**Figure 2:**
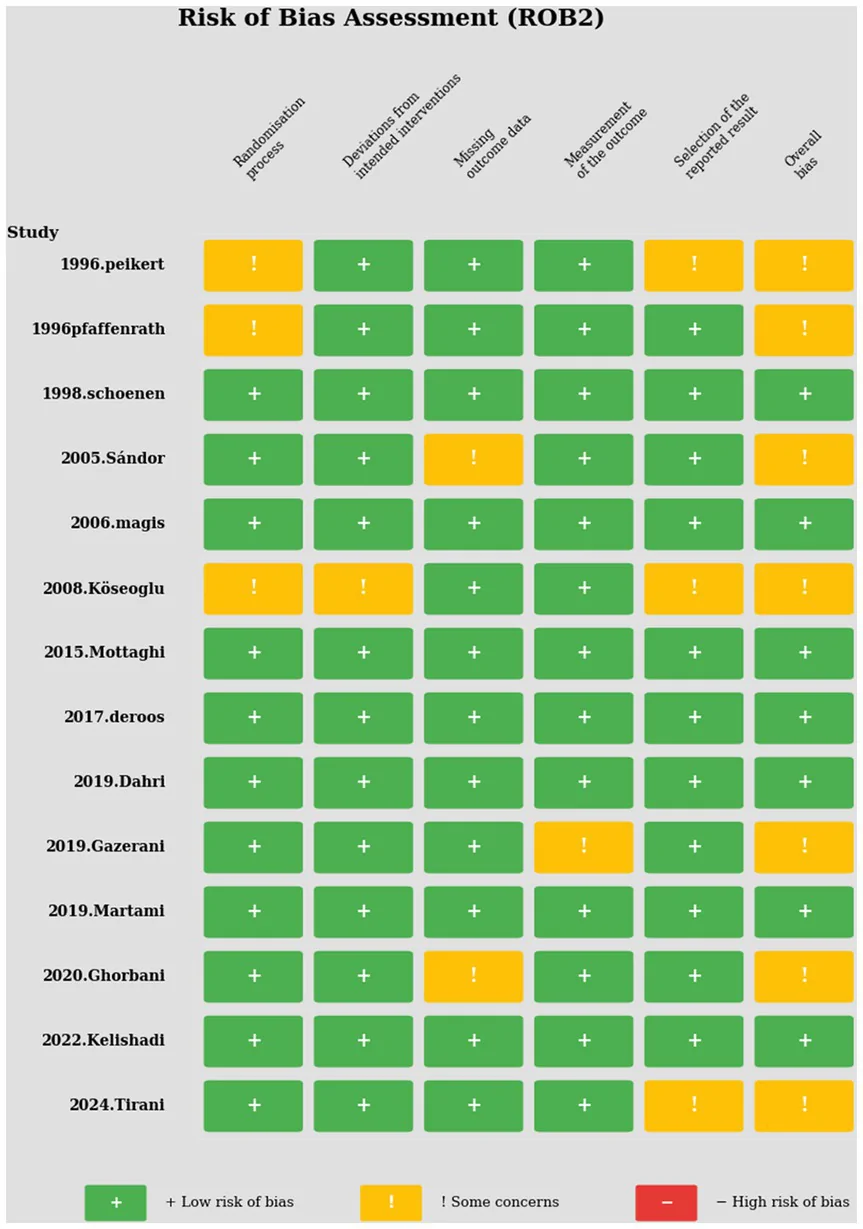
Risk of bias assessment (ROB2). Summary: Low risk of bias: 7 studies (50%); some concerns: 7 studies (50%); high risk of bias: 0 studies (0%). Most concerns arose from incomplete reporting of randomization procedures in older studies and selective reporting of results. No studies were judged to have high risk of bias overall.

Figure 2 presents the risk-of-bias assessment for the 14 studies included in the primary analysis. Seven studies were assessed as having a low risk of bias, seven were classified as having some concerns, and none were rated as having a high risk of bias. Most concerns arose from incomplete reporting of randomization procedures in older studies and potential selective reporting.

### 3.3 Network meta-analysis results

#### 3.3.1 Migraine duration

**Figure 3:**
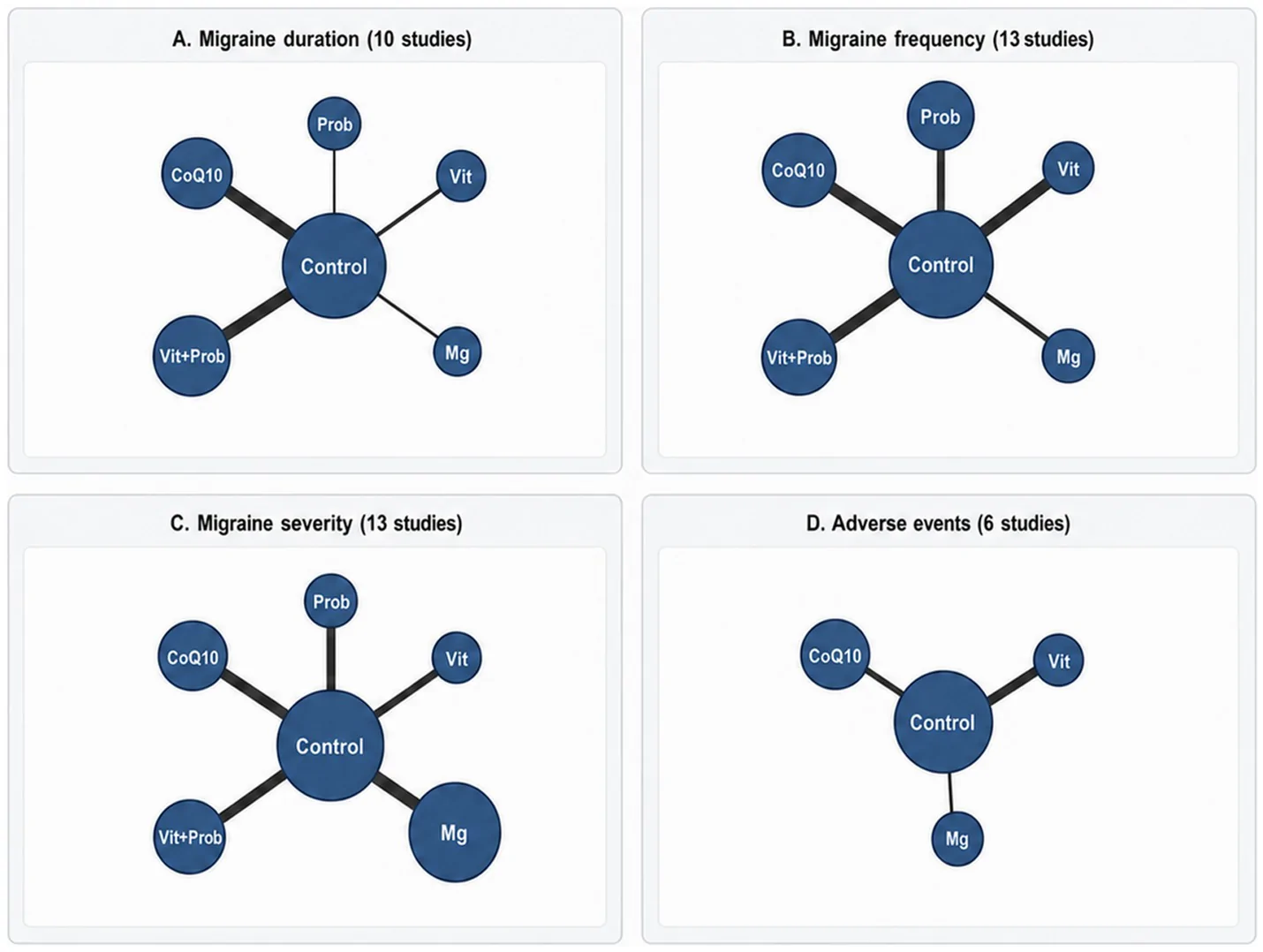
Network evidence plots for different outcomes. Network evidence plots showing treatment comparison networks for different outcome measures. Node size is proportional to the number of participants, and edge thickness represents the number of direct comparisons. **(A)** Migraine duration network (10 studies) showing CoQ10 and vitamin plus probiotics as key interventions, **(B)** Migraine frequency network (13 studies) with probiotics as the most connected intervention, **(C)** Migraine severity network (13 studies) highlighting magnesium and CoQ10 connections, **(D)** Adverse events network (6 studies) with limited comparisons. CoQ10, Coenzyme Q10; Vit, Vitamins; Prob, Probiotics; Vit + Prob, Vitamin plus probiotics; Mg, Magnesium.

**Figure 4:**
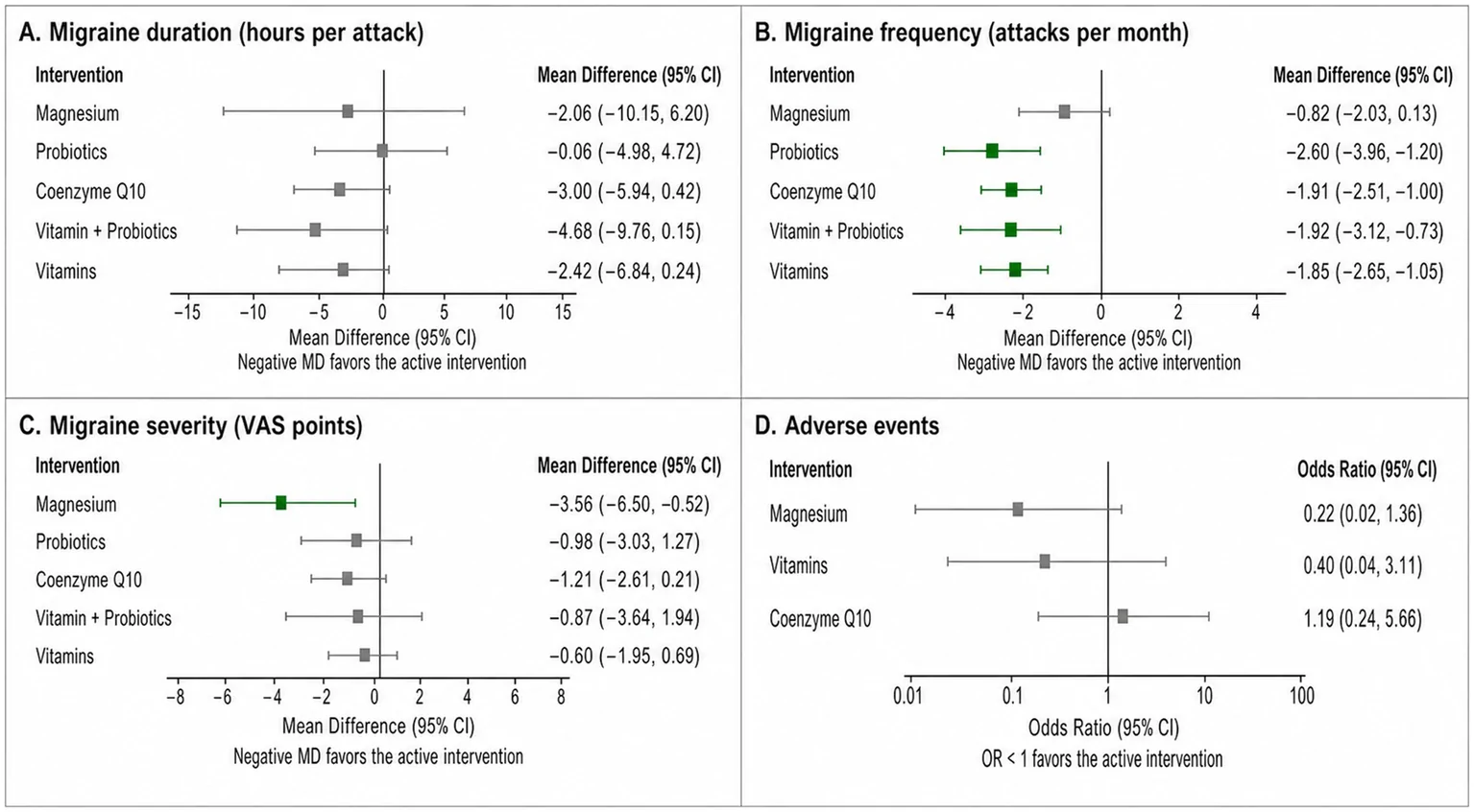
Forest plots of network meta-analysis results. Forest plots showing network meta-analysis results for different outcome measures compared to control/placebo. Effect sizes are presented as mean differences (MD) for continuous outcomes and odds ratios (OR) for adverse events, with 95% confidence intervals. Green squares indicate statistically significant effects (CI does not cross null line), while gray squares indicate non-significant effects. **(A)** Migraine duration: Vit + Prob showed the largest reduction but was not statistically significant, **(B)** Migraine frequency: Probiotics demonstrated the most significant reduction, followed by vitamin plus probiotics, CoQ10, and vitamins, **(C)** Migraine severity: Only magnesium showed statistically significant improvement, **(D)** Adverse events: No statistically significant difference was observed between any active intervention and control. CoQ10, Coenzyme Q10; Vit + Prob, Vitamin plus probiotics.

Ten studies reported migraine-duration data (Figure 3A). Compared with control, vitamin plus probiotics showed the largest estimated reduction in migraine duration (MD = −4.68 h per attack, 95% CI: −9.76 to 0.15), followed by coenzyme Q10 (MD = −3.00 h, 95% CI: −5.94 to 0.42), vitamins (MD = −2.42 h, 95% CI: −6.84 to 0.24), magnesium (MD = −2.06 h, 95% CI: −10.15 to 6.20), and probiotics (MD = −0.06 h, 95% CI: −4.98 to 4.72; Figure 4A).

Vitamin plus probiotics had the highest probability of ranking first at 59%, followed by magnesium at 22%, coenzyme Q10 and vitamins at approximately 9% each, and probiotics at 2%.

Critically, the confidence intervals for all active interventions crossed the null value. Therefore, no nutritional supplement demonstrated statistically significant superiority over control for reducing migraine duration. The treatment rankings for this outcome should be considered exploratory and hypothesis-generating rather than evidence of clinically established differences between interventions.

Within-network heterogeneity analysis showed I^2^ = 38.1% (*p* = 0.17) for vitamin comparisons, I^2^ = 31.2% (*p* = 0.22) for coenzyme Q10 comparisons, and I^2^ = 25.4% (*p* = 0.28) for magnesium comparisons, suggesting low to moderate heterogeneity. Global I^2^ was 34.5% (*p* = 0.16), and τ^2^ was 0.36 for the overall network, indicating moderate between-study variance.

**Figure 5:**
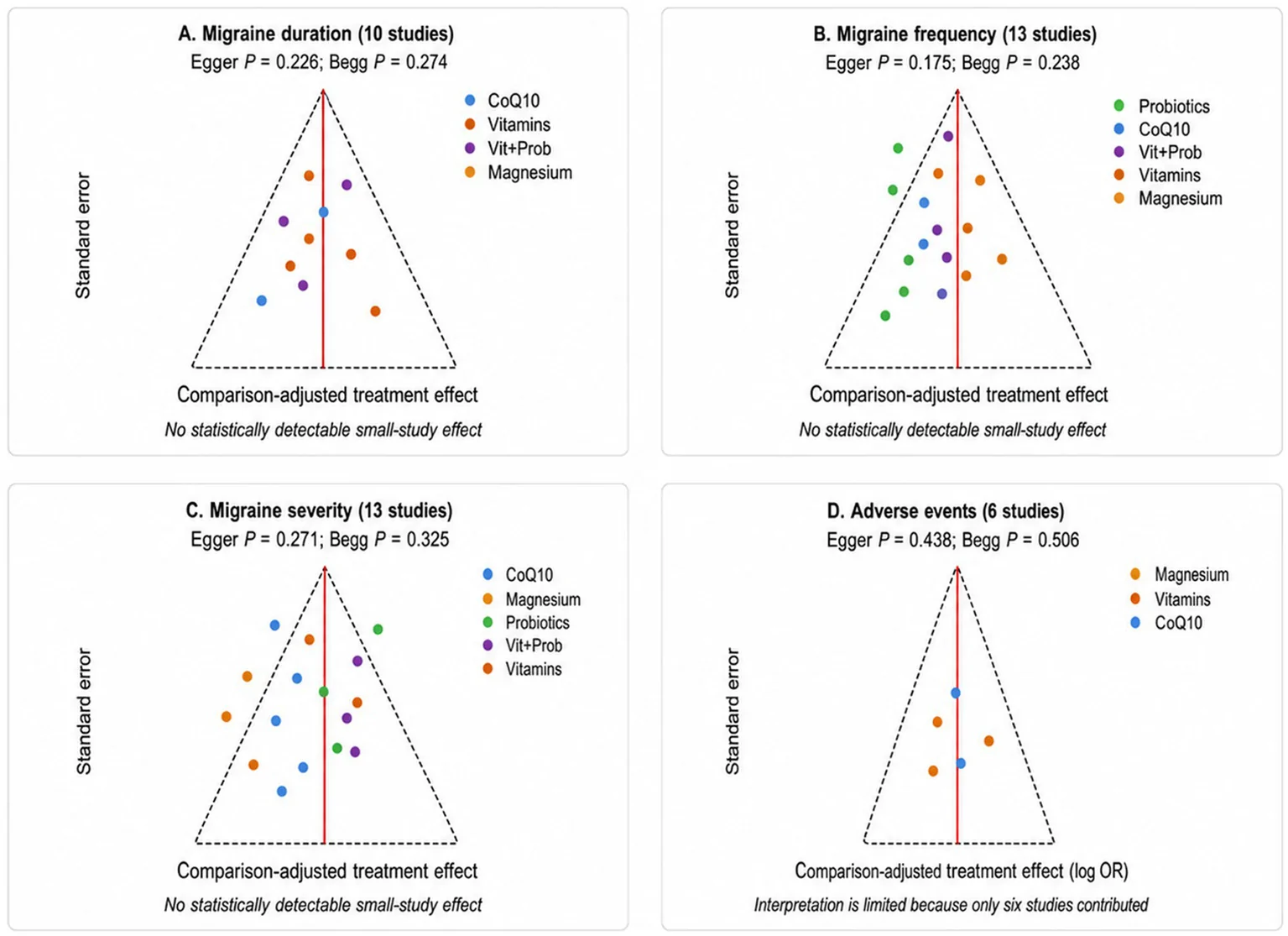
Funnel plots for publication bias assessment. Funnel plots for assessment of publication bias across different outcome measures. Each plot shows the relationship between effect size (x-axis) and standard error (y-axis) for treatment comparisons. The dashed lines represent the 95% confidence intervals, the red vertical line indicates no effect. *p*-values in subplot titles represent Egger’s and Begg’s test results, respectively. **(A)** Migraine duration (10 studies; Egger’s test *p* = 0.226 and Begg’s test *p* = 0.274), **(B)** Migraine frequency (13 studies; Egger’s test *p* = 0.175 and Begg’s test *p* = 0.238), **(C)** Migraine severity (13 studies; Egger’s test *p* = 0.271 and Begg’s test *p* = 0.325), **(D)** Adverse events (6 studies: Egger’s test *p* = 0.438 and Begg’s test *p* = 0.506). CoQ10, Coenzyme Q10; Vit + Prob, Vitamin plus probiotics.

The comparison-adjusted funnel plot for migraine duration is presented in Figure 5A. Neither Egger’s regression test (*p* = 0.226) nor Begg’ s rank-correlation test (*p* = 0.274) indicated a statistically detectable small-study effect. However, because only 10 studies contributed to this outcome, the power of these assessments was limited, and publication bias cannot be excluded.

CINeMA indicated moderate-certainty evidence for migraine duration, downgraded for within-study bias and imprecision.

#### 3.3.2 Migraine frequency

Thirteen studies involving 693 participants reported migraine-frequency data (Figure 3B). Compared with control, probiotics showed the largest estimated reduction in migraine frequency (MD = −2.60 attacks/month, 95% CI: −3.96 to −1.20), followed by vitamin plus probiotics (MD = −1.92 attacks/month, 95% CI: −3.12 to −0.73), coenzyme Q10 (MD = −1.91 attacks/month, 95% CI: −2.51 to −1.00), vitamins (MD = −1.85 attacks/month, 95% CI: −2.65 to −1.05), and magnesium (MD = −0.82 attacks/month, 95% CI: −2.03 to 0.13). The confidence intervals for probiotics, vitamin plus probiotics, coenzyme Q10, and vitamins excluded the null value, whereas the confidence interval for magnesium crossed the null value (Figure 4B).

Probiotics had the highest probability of ranking first at 72%, followed by vitamin plus probiotics at 13%, vitamins at 8%, coenzyme Q10 at 7%, and magnesium at approximately 1%. Minor differences in the sum of these probabilities reflect rounding.

None of the comparisons between active interventions achieved statistical significance. Therefore, although probiotics had the most favorable point estimate and the highest probability of ranking first, the available evidence did not demonstrate that probiotics were statistically superior to any other active supplement category.

Within-network heterogeneity analysis showed I^2^ = 15.4% (*p* = 0.32) for probiotic comparisons, I^2^ = 41.2% (*p* = 0.11) for coenzyme Q10 comparisons, I^2^ = 46.8% (*p* = 0.07) for vitamin comparisons, and I^2^ = 28.5% (*p* = 0.25) for magnesium comparisons. Global I^2^ was 37.3% (*p* = 0.14), and τ^2^ was 0.32 for the overall network.

The comparison-adjusted funnel plot for migraine frequency is presented in Figure 5B. Neither Egger’s regression test (*p* = 0.175) nor Begg’s rank-correlation test (*p* = 0.238) indicated a statistically detectable small-study effect. Nevertheless, the nonsignificant results should not be interpreted as evidence that publication bias was absent because these tests may have limited power in an evidence base of 13 studies.

The network was predominantly star-shaped, with active interventions connected mainly through the placebo or control node. Formal node-splitting was therefore restricted to the few comparisons for which closed loops were available. No statistically detectable inconsistency was identified in the assessable loops. However, inconsistency tests have limited power in sparse networks. These findings indicate an absence of detectable inconsistency rather than proof that the entire network was consistent.

CINeMA assessment revealed moderate certainty evidence for migraine frequency, downgraded for within-study bias (methodological limitations in randomization and allocation concealment in some studies) and reliance on indirect evidence.

#### 3.3.3 Migraine severity

Thirteen studies reported migraine-severity data using a 10-point visual analog scale (Figure 3C). Compared with control, magnesium showed the largest reduction in migraine severity (MD = −3.56 points, 95% CI: −6.50 to −0.52), followed by coenzyme Q10 (MD = −1.21 points, 95% CI: −2.61 to 0.21), probiotics (MD = −0.98 points, 95% CI: −3.03 to 1.27), vitamin plus probiotics (MD = −0.87 points, 95% CI: −3.64 to 1.94), and vitamins (MD = −0.60 points, 95% CI: −1.95 to 0.69; Figure 4C).

Magnesium had the highest probability of ranking first at 85%, followed by vitamin plus probiotics at 6%, probiotics at 4%, coenzyme Q10 at 3%, and vitamins at 1%. Magnesium was the only intervention whose confidence interval excluded the null value.

Although magnesium had both the most favorable point estimate and the highest probability of ranking first, ranking probabilities should not be interpreted as standalone evidence of comparative superiority. The limited number of contributing studies means that the magnitude and stability of the estimated benefit require further confirmation.

Within-network heterogeneity analysis showed I^2^ = 42.5% (*p* = 0.09) for coenzyme Q10 comparisons, I^2^ = 35.2% (*p* = 0.15) for vitamin comparisons, I^2^ = 29.6% (*p* = 0.22) for magnesium comparisons, and I^2^ = 21.3% (*p* = 0.29) for probiotic comparisons. Global I^2^ was 38.6% (*p* = 0.12), and τ^2^ was 0.39 for the overall network.

The comparison-adjusted funnel plot for migraine severity is presented in Figure 5C. Neither Egger’s regression test (*p* = 0.271) nor Begg’s rank-correlation test (*p* = 0.325) indicated a statistically detectable small-study effect. However, these findings should be interpreted cautiously and do not exclude publication bias.

No statistically detectable disagreement between direct and indirect evidence was identified in the assessable loops. However, the limited network structure reduced the power of inconsistency assessment.

CINeMA assessment revealed moderate certainty evidence for migraine severity, downgraded for heterogeneity and imprecision due to small sample sizes.

#### 3.3.4 Adverse events

Six studies reported adverse event rates (Figure 3D). Network meta-analysis showed that compared to control, magnesium had the lowest risk of adverse events [OR = 0.22, 95%CI: (0.02, 1.36)], followed by vitamins [OR = 0.40, 95%CI: (0.04, 3.11)] and coenzyme Q10 [OR = 1.19, 95%CI: (0.24, 5.66)] (Figure 4D).

Magnesium had the most favorable safety ranking. However, the confidence intervals for all interventions included the null value, indicating no statistically significant differences between groups.

The global I^2^ was 16.4%, and τ^2^ was 0.18.

Only six studies contributed adverse-event data; therefore, visual interpretation of the comparison-adjusted funnel plot and formal assessment of small-study effects were considered unreliable (Figure 5D). Egger’s regression test (*p* = 0.438) and Begg’s rank-correlation test (*p* = 0.506) were reported for completeness, and neither was statistically significant. Given the very small number of contributing studies, these results cannot exclude small-study effects or publication bias.

CINeMA assessment revealed low to moderate certainty evidence for adverse events, downgraded for within-study bias (limited number of studies reporting this outcome) and imprecision (low event rates and wide confidence intervals).

### 3.4 Network meta-regression analysis

For migraine frequency, intervention duration was identified as a statistically significant study-level effect modifier (coefficient = −0.09, 95% CI: −0.17 to −0.02; *p* = 0.01), indicating that longer intervention durations were associated with larger reductions in migraine frequency. Baseline migraine frequency showed a trend toward association (coefficient = −0.14, 95% CI: −0.29 to 0.01; *p* = 0.07). Mean age, proportion of female participants, publication year, and sample size were not statistically significant effect modifiers. Because this analysis was based on aggregate study-level covariates and a relatively small number of studies, it should not be interpreted as establishing an individual-level causal relationship.

For migraine severity, none of the prespecified covariates reached statistical significance. Baseline migraine frequency showed a borderline association, while intervention duration, mean age, and publication year showed no statistically significant effects.

For migraine duration, intervention duration approached significance (coefficient = −0.12, 95%CI: −0.25 to 0.01; *p* = 0.06), while the other covariates showed no statistically significant associations.

### 3.5 Trial sequential analysis

After exclusion of Shoeibi et al. 2017, the direct evidence contributing to the migraine-frequency network was reassessed before TSA. The direct probiotic-versus-control evidence consisted of one trial involving 40 participants, and the vitamin-plus-probiotics comparison consisted of one trial involving 72 participants. TSA was therefore not used to draw evidence-sufficiency conclusions for these single-trial comparisons.

**Figure 6:**
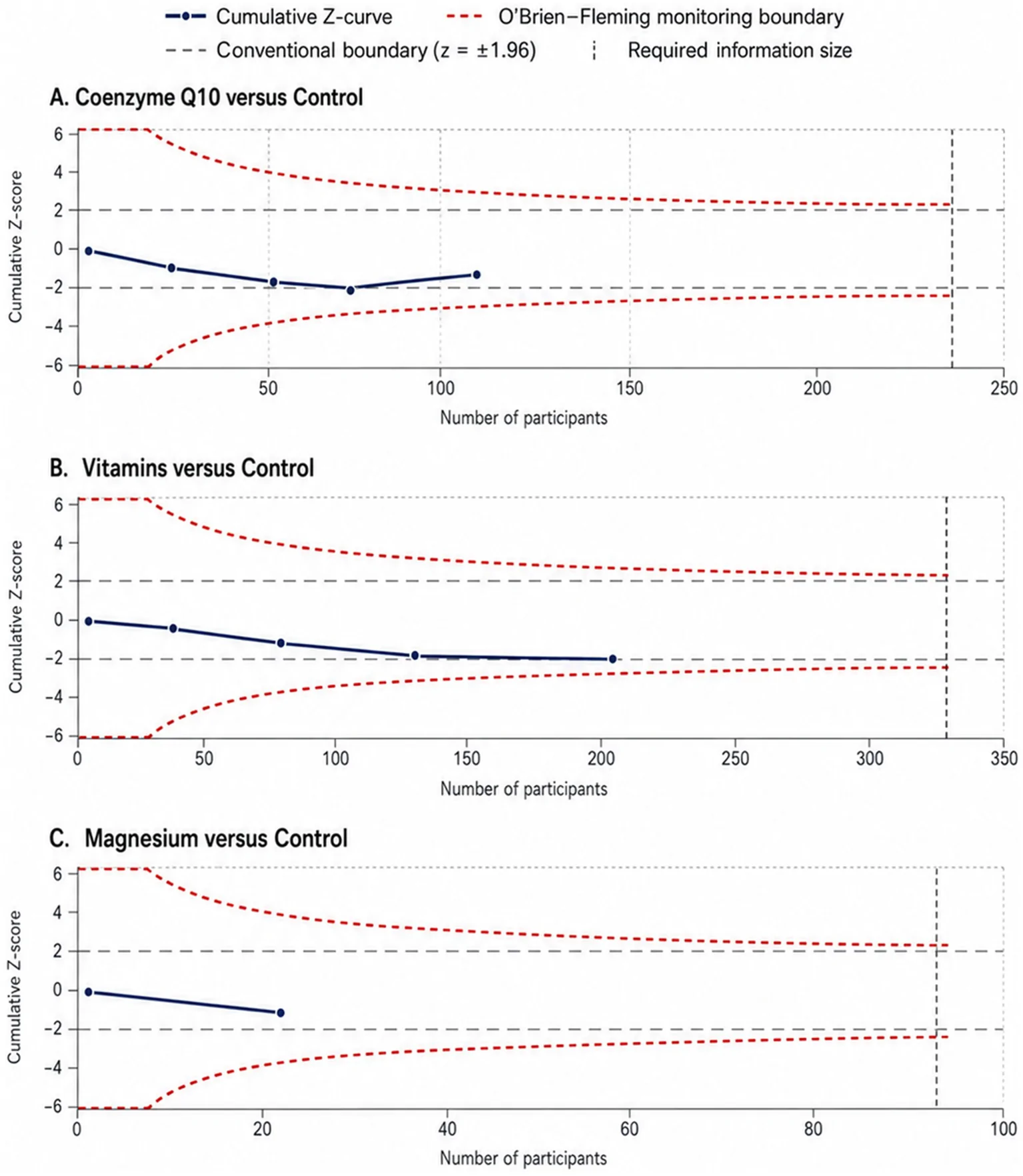
Trial sequential analysis for migraine frequency (attacks per month). Trial sequential analysis for migraine frequency. The cumulative *Z*-curve (solid blue with trial markers) is plotted against O’Brien–Fleming-type *α*-spending monitoring boundaries (dashed red) and conventional two-sided significance boundaries at *z* = ±1.96 (dashed gray). The vertical dashed line indicates the diversity-adjusted required information size (IS). Panel **A** presents coenzyme Q10 versus control based on four studies involving 210 participants, the cumulative direct-evidence *Z*-curve did not cross the trial sequential monitoring boundary and remains inconclusive. Panel **B** presents vitamins versus control based on five studies involving 290 participants, the cumulative direct-evidence *Z*-curve did not cross the trial sequential monitoring boundary and remains inconclusive. Panel **C** presents magnesium versus control based on two studies involving 81 participants the cumulative direct-evidence *Z*-curve did not cross the trial sequential monitoring boundary, and remains inconclusive.

The updated direct evidence included four coenzyme Q10-versus-control studies involving 210 participants, five vitamins-versus-control studies involving 290 participants, and two magnesium-versus-control studies involving 81 participants. TSA was conducted for these comparisons using the corresponding updated datasets. Given the limited accrued information and sensitivity of monitoring boundaries to the analytical assumptions, no result was described as establishing conclusive or definitive efficacy (Figures 6A–C).

These TSA findings apply only to the corresponding direct pairwise comparisons and cannot be extrapolated to indirect comparisons or treatment rankings generated by the network meta-analysis.

### 3.6 Sensitivity analyses

Sensitivity analyses excluding studies assessed as having some concerns for risk of bias, restricting the evidence base to double-blind placebo-controlled trials, using standardized mean differences instead of mean differences, excluding older studies, and conducting leave-one-out analyses produced broadly similar directions of effect and did not materially change the principal treatment hierarchy. Standardized mean differences were used only in the corresponding sensitivity analysis and were treated as dimensionless effect measures.

Shoeibi et al. was not included in any sensitivity analysis because it did not meet the eligibility framework applied to the revised quantitative synthesis.

### 3.7 Subgroup analyses

Subgroup analyses based on intervention duration and baseline migraine frequency suggested possible differences in treatment effects across study groups. However, no statistically significant subgroup interaction was identified. These results should be considered exploratory because few studies contributed to each subgroup and the analyses were based on aggregate study-level characteristics.

## 4 Discussion

This network meta-analysis incorporated network meta-analysis, TSA restricted to eligible direct pairwise evidence, and network meta-regression to evaluate nutritional supplements for migraine prevention. The updated frequency analysis included 13 studies involving 693 participants. Probiotics showed the largest estimated reduction in migraine frequency and the highest probability of ranking first, followed by vitamin plus probiotics, coenzyme Q10, and vitamins. However, none of the comparisons between active interventions was statistically significant. Vitamin plus probiotics showed the largest estimated reduction in migraine duration without reaching statistical significance, while magnesium showed the largest reduction in migraine severity. These findings should be interpreted cautiously because the network was predominantly star-shaped, most active-treatment comparisons were indirect, and several intervention nodes included clinically heterogeneous formulations.

Treatment rankings should be interpreted cautiously in this small and predominantly star-shaped network. Rank probabilities and SUCRA values summarize different features of the relative treatment ordering and should not be used interchangeably. Moreover, neither ranking measure adequately reflects the precision of the corresponding treatment estimate. Clinical interpretation should therefore prioritize effect estimates, confidence intervals, direct-evidence availability, and certainty assessments over rankings alone.

An important structural limitation is the predominantly star-shaped topology of the network. Active supplements were connected mainly through the common placebo or control node, with very limited direct head-to-head comparisons. Consequently, most comparisons between active interventions were based on indirect evidence and depended on the transitivity assumption. Although no statistically detectable inconsistency was identified, the available inconsistency tests had limited power. Treatment rankings should therefore be regarded as hypothesis-generating, and direct head-to-head trials are required to establish comparative effectiveness.

The findings for migraine frequency are partly consistent with previous meta-analyses. Talandashti et al. reported favorable effects of magnesium and coenzyme Q10 (16), while Ketata and Ellouz reported favorable rankings for selected nutraceuticals (17).

The present study additionally evaluated probiotics and vitamin-probiotic combinations as separate intervention categories.

Probiotics showed the largest estimated reduction in migraine frequency compared with control (MD = −2.60 attacks/month, 95% CI: −3.96 to −1.20) and had a 72% probability of ranking first. Vitamin plus probiotics, coenzyme Q10, and vitamins also demonstrated statistically significant reductions compared with control. However, no comparison between active interventions achieved statistical significance. The favorable ranking of probiotics therefore does not establish direct comparative superiority over other supplement categories.

The predominantly star-shaped network is particularly important when interpreting the frequency results. Comparisons between probiotics and the other active supplements were derived through the common control node rather than direct head-to-head evidence. The probiotic estimate and ranking consequently depend on the transitivity assumption and may change when additional direct trials become available.

The updated direct-evidence structure substantially changed the interpretation of TSA. Only one direct probiotic-versus-control trial involving 40 participants contributed to the migraine-frequency dataset. Consequently, the previous TSA-based claim that the evidence for probiotics crossed a monitoring boundary after three trials and 312 participants was withdrawn. No TSA-based evidence-sufficiency conclusion was drawn for probiotics from a single direct trial.

TSA was retained only for comparisons represented by at least two direct trials. Even where conventional statistical significance may be observed, TSA findings should be interpreted cautiously because accrued information remains limited and monitoring boundaries are sensitive to the prespecified clinically relevant difference, heterogeneity, and error assumptions. TSA does not provide information about the reliability of indirect network comparisons or treatment rankings.

Intervention duration was identified as a potential study-level effect modifier for migraine frequency, with each additional week of treatment associated with an estimated reduction of 0.09 attacks per month. However, because the finding was derived from aggregate study-level meta-regression, it should not be interpreted as establishing a causal individual-level relationship or a specific minimum treatment duration.

The preliminary finding that probiotics may reduce migraine frequency is consistent with the proposed involvement of the gut–brain axis in migraine pathophysiology. Nevertheless, the frequency estimate for the probiotics node was informed by only one direct probiotic-versus-control study in the updated dataset. The favorable network estimate and ranking therefore depend substantially on indirect evidence and should not be generalized to all probiotic strains, doses, or commercially available products. Mechanistic plausibility does not establish clinical efficacy, and additional placebo-controlled and head-to-head trials are required.

Coenzyme Q10 has biological plausibility through its involvement in mitochondrial energy metabolism and oxidative-stress regulation (7). However, the updated network analysis did not demonstrate a statistically significant effect on migraine severity (MD = −1.21 points, 95% CI: −2.61 to 0.21). The findings therefore do not support a definitive conclusion regarding its effect on migraine severity.

Vitamin plus probiotics showed the largest estimated reduction in migraine duration, but its confidence interval crossed the null value (MD = −4.68 h per attack, 95% CI: −9.76 to 0.15). This result should therefore be interpreted as exploratory rather than evidence of an established synergistic effect. Further trials are required to determine whether combined vitamin and probiotic supplementation provides clinically meaningful benefits beyond either intervention alone.

Magnesium showed the largest estimated reduction in migraine severity and was the only intervention whose confidence interval excluded the null value for this outcome (MD = −3.56 points, 95% CI: −6.50 to −0.52). It also showed a favorable adverse-event estimate. Nevertheless, the adverse-event comparison was not statistically significant, and the amount of direct evidence remained limited. These findings suggest a potential benefit but are insufficient to establish definitive comparative superiority or recommend a particular magnesium formulation or dose.

The updated statistical heterogeneity was low to moderate across the three primary outcomes. Global I^2^ values were 34.5% for duration, 37.3% for frequency, and 38.6% for severity, with corresponding τ^2^ values of 0.36, 0.32, and 0.39. Although these values do not indicate substantial statistical heterogeneity, they do not eliminate concerns regarding clinical heterogeneity arising from differences in formulations, doses, intervention durations, populations, and outcome measurements.

The moderate evidence quality identified through our assessment reflects inherent limitations in the available literature rather than fundamental flaws in study design or conduct. Many included trials were conducted as pilot or feasibility studies with relatively small sample sizes, reflecting the evolving understanding of nutritional interventions in migraine management. Additionally, the complexity of measuring subjective outcomes such as pain severity and functional disability introduces measurement variability that may attenuate observed effect sizes.

Comparison-adjusted funnel plots and formal statistical tests did not identify statistically detectable small-study effects for migraine duration, frequency, severity, or adverse events. However, the statistical power of Egger’s and Begg’s tests was limited by the modest number of studies contributing to each outcome, particularly adverse events, for which only six studies were available. Therefore, nonsignificant results should not be interpreted as demonstrating the absence of publication bias.

If confirmed by larger direct head-to-head trials, these findings may contribute to individualized nutritional approaches to migraine prevention. At present, however, the evidence is insufficient to recommend one supplement category over another solely on the basis of treatment rankings. Clinical decisions should consider evidence certainty, patient preferences, comorbidities, potential interactions, tolerability, formulation quality, and established preventive therapies.

This study has several limitations that should be acknowledged. First, the relatively small number of included trials (14 RCTs, 791 participants) compared with broader reviews in the field (16, 18) limits the precision of our effect estimates and the statistical power of subgroup analyses. This smaller sample reflects our focus on specific supplement categories including probiotics and combination therapies, which have a more limited evidence base than well-established interventions. Second, the limited number of head-to-head comparisons among nutritional supplements necessitates continued reliance on indirect comparisons, which, while statistically valid, cannot fully replace direct comparative evidence. Third, the predominant focus on short-term outcomes in existing studies limits understanding of long-term efficacy and safety profiles, which are crucial considerations for chronic conditions requiring extended treatment.

A further methodological limitation was the pragmatic grouping of clinically and mechanistically different supplements into broad intervention nodes. Although this strategy maintained network connectivity, it may have introduced within-node clinical heterogeneity and reduced the clinical specificity of the pooled estimates. Individual vitamins, magnesium salts, and probiotic formulations may differ in biological targets, bioavailability, dosage, bacterial composition, colony-forming unit counts, viability, and clinical effects. Results should therefore be interpreted at the supplement-category level and should not be extrapolated directly to a specific product, formulation, strain, or dose.

Differences in intervention dosage, treatment adherence, adherence monitoring, manufacturing quality, and supplement standardization may also have influenced the observed effects. These factors could attenuate or inflate pooled estimates and limit the generalizability of the findings to commercially available nutritional supplements.

The application of TSA was restricted to direct pairwise comparisons because TSA has not been validated for indirect or mixed treatment estimates derived from network meta-analysis. Comparisons represented by only one direct trial were not used to draw evidence-sufficiency conclusions. Consequently, TSA findings pertain only to the corresponding direct evidence and cannot determine the reliability of network-derived indirect comparisons or treatment rankings.

Future trials should use adequate sample sizes, standardized outcome definitions, longer follow-up periods, consistent reporting of adverse events, and direct head-to-head comparisons among the most promising interventions.

## 5 Conclusion

Probiotics showed the largest estimated reduction in migraine frequency and the highest probability of ranking first, while magnesium showed a potential benefit for reducing migraine severity. Vitamin plus probiotics showed the largest estimated reduction in migraine duration, but no intervention demonstrated statistically significant superiority over control for this outcome. The favorable ranking of probiotics should not be interpreted as definitive comparative superiority because no active-treatment comparison was statistically significant and most comparisons depended on indirect evidence through control. Only one direct probiotic-versus-control trial contributed to the updated frequency dataset; therefore, no TSA-based evidence-sufficiency conclusion was drawn for probiotics. Treatment rankings should be regarded as exploratory. Larger, adequately powered, multicenter randomized controlled trials with standardized outcomes, longer follow-up, and direct head-to-head comparisons are required before firm clinical recommendations can be made.

## Data Availability

The original contributions presented in the study are included in the article/Supplementary material, further inquiries can be directed to the corresponding author.
