# Supplementary material for: Nutritional supplements for migraine prevention: evaluating evidence sufficiency and effect modifiers through network meta-analysis, trial sequential analysis, and meta-regression
Source: Front Nutr. 2026 Aug 24;13:1911863. doi: 10.3389/fnut.2026.1911863 (PMC13547464; doi:10.3389/fnut.2026.1911863)
Supplement: Supplementary file 2 [file Table_2.DOCX]

**Supplementary file 2:**

**Search strategy:**

**PubMed/Medline:**

#1 "Migraine Disorders"[Mesh] OR migraine*[Title/Abstract] OR headache*[Title/Abstract]

#2 "Nutritional Supplements"[Mesh] OR "Diet Therapy"[Mesh] OR "Micronutrients"[Mesh] OR supplement*[Title/Abstract] OR diet*[Title/Abstract] OR nutrient*[Title/Abstract] OR vitamin*[Title/Abstract] OR "Magnesium"[Title/Abstract] OR "Riboflavin"[Title/Abstract] OR "Coenzyme Q10"[Title/Abstract] OR "Omega-3"[Title/Abstract] OR "Probiotics"[Title/Abstract]

#3 "Randomized Controlled Trial"[Publication Type] OR "randomized"[Title/Abstract] OR "placebo"[Title/Abstract] OR "controlled clinical trial"[Title/Abstract] OR "trial"[Title/Abstract]

#4 #1 AND #2 AND #3

EMBASE

#1 'migraine'/exp OR migraine*:ti,ab OR headache*:ti,ab

#2 'nutritional supplement'/exp OR 'diet therapy'/exp OR 'micronutrient'/exp OR supplement*:ti,ab OR diet*:ti,ab OR nutrient*:ti,ab OR vitamin*:ti,ab OR 'magnesium':ti,ab OR 'riboflavin':ti,ab OR 'coenzyme Q10':ti,ab OR 'omega-3':ti,ab OR 'probiotic':ti,ab

#3 'randomized controlled trial'/exp OR randomized:ti,ab OR placebo:ti,ab OR 'controlled clinical trial':ti,ab OR trial:ti,ab

#4 #1 AND #2 AND #3

**Web of Science**

#1 TS=(migraine* OR headache*)

#2 TS=(nutritional supplement* OR diet* OR nutrient* OR vitamin* OR magnesium OR riboflavin OR "coenzyme Q10" OR "omega-3" OR probiotic*)

#3 TS=(randomized OR placebo OR "controlled clinical trial" OR trial)

#4 #1 AND #2 AND #3

**Cochrane Library**

#1 [MeSH descriptor: "Migraine Disorders"] OR migraine*:ti,ab,kw OR headache*:ti,ab,kw

#2 [MeSH descriptor: "Nutritional Supplements"] OR [MeSH descriptor: "Diet Therapy"] OR supplement*:ti,ab,kw OR diet*:ti,ab,kw OR nutrient*:ti,ab,kw OR vitamin*:ti,ab,kw OR magnesium:ti,ab,kw OR riboflavin:ti,ab,kw OR "coenzyme Q10":ti,ab,kw OR "omega-3":ti,ab,kw OR probiotic*:ti,ab,kw

#3 randomized:ti,ab,kw OR placebo:ti,ab,kw OR "controlled clinical trial":ti,ab,kw OR trial:ti,ab,kw

#4 #1 AND #2 AND #3
